# Safety and Efficacy of Infliximab in Severe Alcoholic Hepatitis: A Systematic Review

**DOI:** 10.7759/cureus.5082

**Published:** 2019-07-04

**Authors:** Muhammad B Majeed, Rohit Agrawal, Bashar M Attar, Yazan Abu Omar, Seema R Gandhi

**Affiliations:** 1 Internal Medicine, John H. Stroger, Jr. Hospital of Cook County, Chicago, USA; 2 Gastroenterology and Hepatology, John H. Stroger, Jr. Hospital of Cook County, Chicago, USA

**Keywords:** infliximab, alcoholic hepatitis

## Abstract

Severe alcoholic hepatitis (SAH) is associated with significant morbidity and mortality, yet the treatment options available are very limited. Past studies have evaluated the efficacy of infliximab in such patients; however, they were limited by sample size. Our aim was to perform a systematic review of these studies to assess the role of infliximab in patients with SAH.

We conducted a literature search using electronic database engines including Ovid, PubMed, Scopus, MEDLINE and Cochrane Library from inception to October 2018 to identify published articles addressing outcomes in patients treated for alcoholic hepatitis with infliximab. The primary outcome reviewed was one-month mortality. Secondary outcomes included rate and type of infection; cause of mortality; levels of aspartate aminotransferase, alanine aminotransferase, bilirubin and tumor necrosis factor-α; and Maddrey discriminant function.

Five studies including two randomized controlled trials and three case series were included in our analysis with a total sample size of 70 patients. One-month mortality ranged from 10% to 17% in patients who received a single dose of infliximab with or without prednisone compared to 38% in patients who received three doses of infliximab in combination with prednisone. A single dose of infliximab was associated with an infection rate of 10% to 26% in contrast to an 89% rate with three doses of infliximab.

Infliximab, when used in a single dose, could potentially be an alternative agent for the management of SAH in a large group of patients who have contraindications such as gastrointestinal bleeding, uncontrolled diabetes or an active hepatitis infection. It might also have a role in the prevention of hepatorenal syndrome. There is a need for larger trials to evaluate the role of infliximab in a cohort of patients who are not candidates for prednisolone therapy.

## Introduction and background

Alcoholic liver disease (ALD) encompasses a spectrum of pathologies, ranging from simple steatosis to frank cirrhosis. Alcoholic hepatitis (AH), a severe manifestation of ALD is caused by excessive alcohol use. The true prevalence of AH is unknown; however, it accounted for 0.8% of all hospitalizations in 2010 in the United States [1]. Despite the increasing prevalence and severity of AH, there are no consistent recommendations for its management.

Severe alcoholic hepatitis (SAH) is defined by a Maddrey discriminant fraction (MDF) of >32 or model for end-stage liver disease (MELD) score of >20, which carries a 28-day mortality ranging from 30 to 50% [2-3]. Various proinflammatory cytokines have been implicated in the pathogenesis of AH including tumor necrosis factor-alpha (TNF-α), interleukin 1 and interleukin 8 (IL-1, IL-8) [4-6]. 

The use of immunosuppressive agents to minimize the proinflammatory state is currently the mainstay of treatment. Prednisolone is considered the standard therapy for patients with AH, when the MDF > 32. However, sepsis, uncontrolled diabetes mellitus and gastrointestinal bleeding are relative contraindications to the use of these corticosteroids, even with a high MDF score [3]. In addition, approximately 40% of patients who receive steroids do not respond as indicated by the Lille score >0.45 after one week [7]. Pentoxifylline, which inhibits the synthesis of the proinflammatory cytokine TNF-α, is another alternative, but it has recently been disproven as an effective therapy [3].

Infliximab, a TNF-α inhibitor, is a treatment modality for chronic inflammatory diseases such as rheumatoid arthritis, psoriatic arthritis, inflammatory bowel disease and ankylosing spondylitis. It has also been used for the management of the above-mentioned chronic inflammatory states in the presence of hepatitis B and C, as well as in liver transplant patients [8]. We conducted a systematic review of the literature to investigate the effectiveness of the therapeutic use of infliximab in patients with SAH. 

## Review

Materials and methods

We conducted a literature search using electronic database engines including Ovid, PubMed, Scopus, MEDLINE and Cochrane Library from inception to October 2018 to identify published articles addressing outcomes in patients treated for alcoholic hepatitis with infliximab. We reviewed the reference lists of all eligible studies to identify additional studies. The combination of keywords used was “alcoholic hepatitis” or “infliximab” or “tumor necrosis factor inhibitor” or “TNF-alpha” and “alcoholic liver disease”.

**Figure 1:**
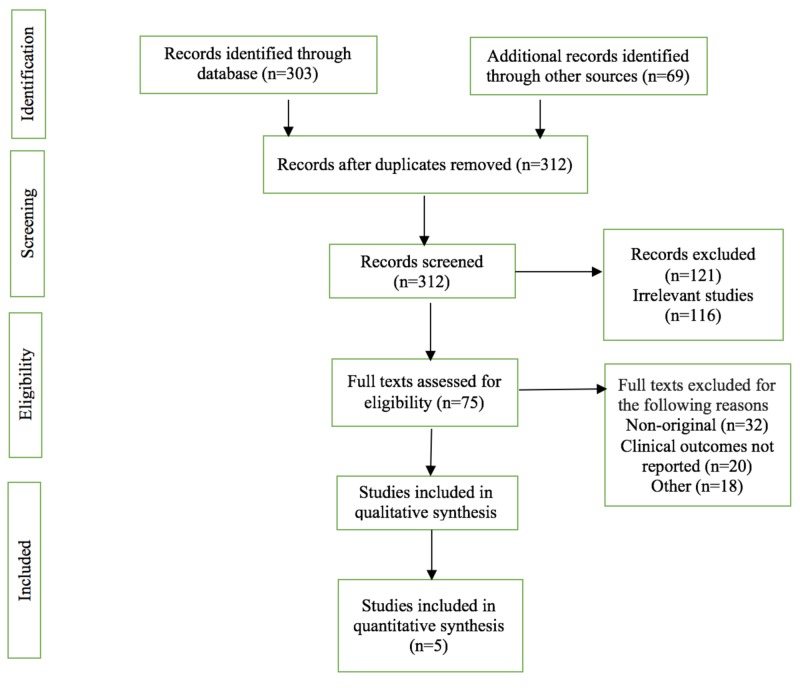
Preferred Reporting Items for Systematic Reviews and Meta-Analyses flow diagram detailing the review process

We included every published study (randomized controlled trials or case series) that reported the use of infliximab for the management of AH. We did not include case reports, abstracts or review articles. Articles without reported outcomes or in a language other than English were also excluded. Two reviewers (MM and RA) independently performed study selection according to the eligibility criteria and using the PRISMA (Preferred Reporting Items for Systematic Reviews and Meta-Analyses) guidelines. Disagreements were resolved by discussion. A PRISMA flow diagram detailing the review process is shown in Figure 1.

We extracted the following variables from the included articles: study characteristics (study design, primary author, time period of study, year of publication and country of the population studied), characteristics of the study population (number of patients, age of patients, gender, Child-Pugh class, MELD score, MDF score, level of aspartate aminotransferase (AST), alanine aminotransferase (ALT), bilirubin and TNF-α).

We used the Newcastle-Ottawa Scale (NOS) and other published criteria to assess the quality of clinical studies included in our systematic review [9-11]. The quality assessment of the collected data was further consolidated by detailed discussion between the two primary investigators (MM and RA) and any disagreement was resolved by discussion with a senior investigator. Risk of bias in randomized controlled trials (RCTs) was analyzed by the Cochrane criteria [12]. However, only two out of five trials were RCTs and rest were case series. The primary outcome analyzed in this study was mortality at one month from initiation of treatment. The secondary outcomes included rate and type of infection; cause of mortality; and levels of AST, ALT, bilirubin, MDF, and TNF-α at the end of one month. Three out of five studies included in this systematic review were case series, and hence, a meta-analysis could not be performed. Findings were reported in the tables and text, as data could not be pooled in forest plots.

Results

The search strategy described above retrieved a total of 312 published articles. Among these, only five trials were identified to qualify for inclusion, as described in Figure 1. The characteristics of these primary studies are detailed in Table 1. 

**Table 1:** Characteristics of the primary studies included in the systematic review RCT, randomized controlled trial

	Sharma et al. [13]	Tilg et al. [14]	Mookerjee et al. [15]	Spahr et al. [16]	Naveau et al. [17]
Time period	2005-2006	Not reported	Not reported	2001-2002	Not reported
Year of publication	2009	2003	2003	2002	2004
Patient population	Not reported	British	British	Swiss	French
Study design	Case series	Case series	Case series	RCT	RCT
Center (n)	Not reported	2	Not reported	2	19

In this study, we investigated data from 70 patients. All patients received at least one dose of infliximab but steroids were used in combination with infliximab in the trials by Spahr et al. and Naveau et al. [16-17]. Moreover, Naveau et al. gave three doses of infliximab in contrast to other studies [17]. The median age of patients ranged from 40 to 52 years with majority being male. Approximately 18 of 59 the patients (no information on remaining 11 patients) had hepatic encephalopathy at the time of presentation. All patients had an MDF score >32 ranging from 39 to 69 at the time of inclusion. Serum TNF-α level was reported in four of the five trials ranging from 8 to 68 pg/ml and total serum bilirubin ranged from 7 to 20 mg/dl at baseline. The baseline characteristics of patients in the included studies are reported in Table 2.

**Table 2:** The baseline characteristics of patients included in the studies NR, not reported; HE, hepatic encephalopathy; MELD, model for end-stage liver disease; MDF, Maddrey discriminant function; ANC, absolute neutrophil count; AST, aspartate aminotransferase; ALT, alanine aminotransferase; CRP, C-reactive protein; TNF, tumor necrosis factor; PT, prothrombin time; WBC, white blood cell; HVPG, hepatic venous pressure gradient; IL, interleukin

Variables	Sharma et al. [13]	Tilg et al. [14]	Mookerjee et al. [15]	Spahr et al. [16]	Naveau et al. [17]
Infliximab dose (n)	1	1	1	1	3
Prednisone use	No	No	No	Yes	Yes
Number of patients (n)	19	12	10	11	18
Median age (years)	40	50.6	52.7	49	52
Male (%)	100	92	80	100	83
Female (%)	0%	8%	20%	0%	17%
HE (n) %	5 (26%)	4 (33%)	6 (60%)	NR	3 (7%)
Median MELD	25	NR	NR	NR	NR
Child-Pugh score	NR	NR	11.8	11	12
Median MDF	66	48.7	68.9	39	60
Median ANC (n/mm^3^)	6792	11715	NR	NR	NR
Median AST (IU/l)	169	54	NR	NR	39
Median ALT (IU/l)	61	28.6	46	NR	NR
Median serum bilirubin (mg/dl)	19.5	16.84	18.19	7.37	13.45
Median serum albumin (g/dl)	2.8	2.89	NR	NR	2.8
Median serum creatinine (mg/dl)	0.8	NR	0.9	NR	0
Median serum CRP (mg/dl)	34	80.07	86.8	NR	NR
Median TNF-α pg/ml	45	68.3	57	8.1	NR
Mean PT (seconds)	NR	19	21.2	46	36
WBC count (n/l)	NR	16.5	17.5	9.6	NR
HVPG (mmHg)	NR	NR	23.4	19	NR
IL-8 (pg/ml)	NR	NR	76	301	293

We primarily reviewed the mortality rate at one month. The studies that analyzed mortality after a single dose of infliximab reported a one-month mortality ranging from 10% to 17% [13-15]. A mortality of 38% was reported for patients who received a combination of prednisolone and three doses of infliximab [17]. The mortality rate at one month is mentioned in Table 3.

**Table 3:** Mortality rate at one-month follow-up and cause of death MRSA, methicillin-resistant *Staphylococcus aureus*

Variables	Sharma et al. [13]	Tilg et al. [14]	Mookerjee et al.[ 15]	Spahr et al. [16]	^Naveau et al. [17]^
Mortali]ty at 1 month n(%)	2 (11%)	2 (17%)	1(10%)	2 (18%)	7 (38%)
Cause of death					
Renal failure	1	-	-	-	-
Disseminated tuberculosis	1	-	-	-	-
Massive hemorrhage	-	-	-	1	-
Liver failure	-	-	-	1	2
Pneumonia	-	-	1	-	1
Candida septicemia	-	1	-	-	1
MRSA septicemia	-	1	-	-	1
Neisseria meningitidis	-	-	-	-	1
Acute pulmonary edema	-	-	-	-	1

In all four trials that used a single dose of infliximab, rate of infection ranged from 10% to 26%; however, the use of three doses of infliximab was associated with a higher infection rate of 89%. The rate and type of infection is reported in Table 4. 

**Table 4:** Type and rate of infection reported in the included publications MRSA, methicillin-resistant *Staphylococcus aureus*; *E. coli*, *Escherichia coli*

Type of infection	Sharma et al. [13]	Tilg et al. [14]	Mookerjee et al. [15]	Spahr et al. [16]	Naveau et al. [17]
Incidence of infection n (%)	5 (26%)	2 (17%)	1 (10%)	2 (18%)	89%
Pneumonia	3	-	-	1	1
Flare of tuberculosis	2	-	-	-	-
Aspiration pneumonia	-	-	1	-	-
MRSA septicemia	-	1	-	1	1
Candida infection	-	1	-	-	4
Neisseria meningitidis	-	-	-	-	1
E. coli septicemia	-	-	-	-	1

In all the studies, the use of infliximab was associated with a reduction of bilirubin. Median serum creatinine was reported in three of the five trials, and no changes in creatinine were reported at the end of one month. The use of infliximab was associated with a 30% reduction in hepatic venous pulmonary gradient [15]. It was also associated with a reduction in erythrocyte sedimentation rate (ESR), C-reactive protein (CRP), TNF and IL-8. Biochemical markers at one month of follow-up are shown in Table 5. 

**Table 5:** Results of biochemical markers at one-month follow-up *P-value < 0.05 NR, not reported; MELD, model for end-stage liver disease; MDF, Maddrey discriminant function; ANC, absolute neutrophil count; ALT, alanine aminotransferase; AST, aspartate aminotransferase; CRP, C-reactive protein; ESR, erythrocyte sedimentation rate; TNF, tumor necrosis factor; PT, prothrombin time; WBC, white blood cell; HVPG, hepatic venous pressure gradient; IL, interleukin

Variables	Sharma et al. [13]	Tilg et al. [14]	Mookerjee et al. [15]	Spahr et al. [16]	Naveau et al. [17]
Time to follow-up (months)	2	1	1	1	2
MELD	19*	NR	NR	NR	NR
MDF	24*	37.7	42.3	12*	28
ANC (n/mm^3^)	3200*	5985	NR	NR	NR
Median ALT (IU/l)	42	34	NR	NR	NR
Median AST (IU/l)	64*	28*	NR	NR	NR
Median serum bilirubin (mg/dl)	5.3*	7.66*	8.25*	2.54	NR
Median serum albumin (g/dl)	3.2*	3.34	NR	NR	NR
Median serum creatinine mg/dl	0.7	0.9	0.8	NR	NR
CRP (mg/dl)	8*	3.8	3.27 *	NR	NR
ESR (mm/h)	14*	NR	NR	NR	NR
TNF-α (pg/ml)	20	NR	45*	15*	NR
PT (seconds)	NR	18.9*	17.8*	71*	NR
WBC (n/l)	NR	10.5	12.3*	7.9*	NR
Reduction on HVPG (%age)	NR	NR	30%	NR	NR
IL-6 (pg/ml)	NR	NR	37.7 *	4.5*	NR
IL-8 (pg/ml)	NR	NR	55.2*	146*	139

Discussion

Despite high prevalence of the AH, specific therapy targeting AH is not available and current therapeutic modalities involve a combination of alcohol abstinence, nutritional support and pharmacotherapy involving prednisolone and pentoxifylline [18]. Prednisolone is the most accepted therapy; nevertheless, its use is limited by several contraindications such as sepsis, diabetes and gastrointestinal hemorrhage. In addition, approximately 40% of patients who receive steroids do not respond. Current pharmacotherapy has not demonstrated significant long-term mortality benefits, and therefore there is need for further therapeutic interventions to alter the course of this disease [19]. 

After analyzing the one-month mortality of infliximab, we found that a single dose was associated with 11%-18% mortality. The current mainstay of therapy for SAH, prednisolone, has demonstrated a 14% 28-day mortality (STOPAH trial) that is comparable to the mortality associated with a single dose of infliximab [19]. However, the combination of steroids and three doses of infliximab has a significantly higher mortality. This reflects the safety of a single dose of infliximab over three doses and highlights the comparable mortality rates of a single dose of infliximab and prednisolone. Subsequently, infliximab can be considered an alternative to prednisolone in patients with relative contraindications to steroid use including sepsis, diabetes and gastrointestinal hemorrhage. Additionally, viral hepatitis is an absolute contraindication to steroids; therefore, infliximab can be considered as a therapeutic option in such a cohort of patients given its proven efficacy in patients with hepatitis B.

Due to its anti-TNF-α activity, infliximab is associated with improvement in biochemical parameters such as total bilirubin, CRP, ESR, IL-6, IL-8 and absolute neutrophil count. It blocks TNF-mediated increase in vascular permeability and vasodilation preventing leukocyte infiltration and subsequent cell injury [20-22]. Although it is beneficial in reducing inflammation in AH, it also increases the risk of infection in already immunocompromised alcoholic and cirrhotic patients [23-24]. Rate of infection with a single dose of infliximab varies from 10% to 26% and is 89% with three doses of infliximab. However, prednisolone is also associated with a 15% risk of infection that is comparable to the risk associated with a single dose of infliximab. We therefore support the criticisms of using multiple doses of infliximab; however, a single dose of infliximab can be considered equivalent to prednisolone in terms of infection with the current data available [9, 25]. 

Pentoxifylline, a similar anti-TNF agent used in patients with AH, has shown to significantly reduce the development of hepatorenal syndrome [26]. Three of the five trials in our systematic review reported serum creatinine at baseline and at 28 days. In those studies, there was no significant elevation of creatinine at 28 days. Due to its similar mechanism of action, it can be speculated that use of infliximab prevented the development of hepatorenal syndrome in these patients, and therefore may have a role in preventing renal dysfunction. 

Study limitations

There are several limitations to this retrospective study. There is a possibility that we could have missed a relevant study despite the meticulous search of comprehensive literature in the commonly used databases. The small sample size of 70 in the included studies could have led to the under- or overestimation of the findings from the systematic review. The large heterogeneity of study design and population characteristics could also have affected the reported findings and cannot be generalizable.

## Conclusions

Infliximab, when used in a single dose, could potentially be an alternative agent for the management of SAH in patients with contraindications to corticosteroids, such as gastrointestinal bleeding, uncontrolled diabetes or an active hepatitis infection. It may also have a role in the the prevention of hepatorenal syndrome. However, the combined use of infliximab with steroids and the use of multiple doses of infliximab is clearly associated with worse outcomes. There is a need for further randomized controlled trials to evaluate the efficacy and safety of a single dose of infliximab in patients with SAH who have contraindications to steroids.
